# Agreement between magnetic resonance imaging and ultrasonography in deep pelvic endometriosis

**DOI:** 10.1590/1806-9282.20241235

**Published:** 2025-03-31

**Authors:** Mihriban Alkan, Gülsüm Kılıçkap

**Affiliations:** 1 Ankara Bilkent City Hospital, Department of Radiology – Ankara, Türkiye.

**Keywords:** Endometriosis, Ultrasonography, Magnetic resonance imaging, Agreement

## Abstract

**OBJECTIVE:**

Deep pelvic endometriosis is the most common cause of chronic pelvic pain and infertility. Guidelines proposed standardized approaches for the diagnosis of deep pelvic endometriosis with ultrasonography and magnetic resonance imaging; however, knowing the reasons for discrepancy is crucial. We aimed to analyze the agreement between ultrasonography and magnetic resonance imaging for deep pelvic endometriosis findings and provide potential pitfalls and reasons for discordant findings.

**METHODS:**

The study group consists of consecutive patients with deep pelvic endometriosis diagnosed on pelvic (n=1) or transvaginal ultrasonography (n=34) who underwent noncontrast pelvic magnetic resonance imaging. The agreement between the ultrasonography and magnetic resonance imaging was assessed using the prevalence and bias-adjusted kappa statistics. Potential pitfalls and reasons for discordant findings were presented.

**RESULTS:**

The study group consisted of 35 patients with deep pelvic endometriosis. The mean age was 39.5±6.2 years. The most common site of involvement was the rectosigmoid colon (n=34, 97.1%), followed by endometrioma/hemorrhagic cyst (n=32, 91.4%). There was a perfect agreement for endometrioma/hemorrhagic cyst (100%), almost perfect agreement for bladder involvement (PABAK=0.886), and moderate agreement for other sites. The number of uterosacral ligament involvement was lower with ultrasonography than with magnetic resonance imaging. However, due to the impact of gas signals on magnetic resonance imaging imaging, the number and boundaries of rectosigmoid deep pelvic endometriosis were better defined with ultrasonography.

**CONCLUSION:**

The agreement between ultrasonography and magnetic resonance imaging for deep pelvic endometriosis findings varies according to the sites of involvement. Ultrasonography and magnetic resonance imaging are not standalone diagnostic techniques but are complementary to each other. We provided potential diagnostic pitfalls.

## INTRODUCTION

Endometriosis is characterized by the presence of an endometrial gland and stroma outside the uterine cavity. The prevalence has been reported to be approximately from 5 to 20%^1^. According to its localization, subtypes have been defined as superficial peritoneal, ovarian, and deep infiltrative endometriosis. Deep pelvic endometriosis (DPE) may be asymptomatic or may cause dysmenorrhea, dyspareunia, pelvic pain, gastrointestinal and urinary tract symptoms, and infertility. DPE is one of the causes of chronic pelvic pain and is included in the differential diagnosis of pelvic congestion syndrome and pelvic inflammatory disease^2,3^.

Pelvic endometriosis can affect all three compartments of the pelvis. In the anterior compartment, it may affect the vesicouterine pouch, vesicovaginal septum, prevesical area, bladder, and ureters. In the middle compartment, it can affect the uterus, ovaries, fallopian tubes, and uterine ligaments. In the posterior compartment, the affected structures are the rectovaginal pouch, retrocervical area, torus uterinus, uterosacral ligament (USL), rectovaginal septum, rectum, and posterior vaginal fornix.

Ultrasonography (US) is the first-line diagnostic method; however, it has some limitations such as limited field of view and operator dependency. Magnetic resonance imaging (MRI) is a method with high diagnostic accuracy and plays an important role in preoperative decision-making^4,5^.

A standardized approach to the assessment of DPE is crucial. The International Deep Endometriosis Analysis Group (IDEA)^6^ and the European Society for Urogenital Radiology (ESUR)^7^ proposed guidelines for the assessment of DPE with ultrasonography and MRI, respectively.

While the guidelines establish a consistent framework, recognizing the limitations in DPE assessment and pinpointing where US and MRI may disagree is crucial. In this study, using the criteria given in these guidelines, we aimed to analyze the agreement of the findings between US and MRI and provide potential pitfalls and reasons for discordant findings.

## METHODS

The study population was created prospectively. Patients referred for pelvic or transvaginal US from the gynecology and obstetrics outpatient clinic between October 2022 and May 2024 and diagnosed with DPE on US underwent noncontrast pelvic MRI. All consecutive and consenting patients over 18 years of age during the study period were included in the study. This study was approved by the local ethics committee and conducted in accordance with the Helsinki Declaration. Informed consent was obtained from the participants.

The lesions were interpreted using the IDEA and ESUR guidelines^6,7^. Pelvic or transvaginal ultrasonography (TVUS) examinations were performed by a radiologist with 20 years of experience in obstetrics and gynecology.

MRI scans were performed with the GE Signa Pioneer 3T device. The acquisition protocols are shown in Table 1. Intravenous hyoscine butylbromide (Buscopan^®^) was administered before MRI to decrease intestinal peristalsis. No rectal or vaginal gel was used. The mean interval between MRI and US examination was 1–2 months. MRI examinations were evaluated by a radiologist experienced in abdominal radiology, without knowledge of US findings. Foci in the anterior, middle, and posterior compartments that are compatible with endometriosis were recorded.

**Table 1:** Magnetic resonance imaging acquisition protocol.

Parameter	Image sequences				
	T1 weighted	Fat saturated T1 weighted	T2 weighted	Fat saturated T2 weighted	Diffusion weighted
Image plane	Axial	Axial/coronal	Axial/sagittal	Coronal	Axial
TR (msn)	687	781/677	4,588/3,349	4,062	9,595
TE (msn)	9.6	10.8/9.7	106/117	111	67.3
FOV (mm)	32×28.8	32×28.8/30×30	32×28.8/22×22	30×30	36×30.6
Slice thickness (mm)	3	3/3	3/3.5	3	3.9
NEX	320×224	320×256/320×224	320×256/300×300	384×256	128×160

FOV: field of view; NEX: number of excitation; TE: time-to-echo; TR: time-to-repetition.

### Statistical analysis

Categorical data were expressed as frequency and percentages, and continuous variables were expressed as mean and standard deviation. The agreement between US and MRI was analyzed using the kappa statistics. As the conventional kappa statistics were affected by prevalence, prevalence and bias-adjusted kappa (PABAK) statistics were calculated to make a fair comparison. Kappa statistics were assessed as follows: 0.21–40 (fair), 0.41–0.60 (moderate), 0.61–0.80 (substantial), and ≥0.81 (almost perfect agreement). Data were analyzed using Stata v17 (Stata Corp., TX, USA).

## RESULTS

Pelvic (n=1) or transvaginal (n=34) US examination, along with pelvic MRI with endometriosis protocol, were performed on 35 patients referred to our clinic. The mean age was 39.5±6.2 years. The most common findings were unilateral or bilateral endometrioma/hemorrhagic cyst, thickening of the rectum and sigmoid colon wall with iso-hypointense signal, and adhesion between the uterus, ovary, and adjacent colon segments.

The endometrioma/hemorrhagic cyst was observed in 32 patients on both US and MRI. Round ligament (n=7), rectovaginal septum (n=11), and ileal involvement (n=1) were observed only with MRI but not with US. There was an almost perfect agreement for bladder involvement (PABAK=0.886); however, the number of the cases was three, all were observed on MRI, whereas only one on US. The agreement between US and MRI was substantial for hemato-/hydrosalpinx (PABAK=0.714), and moderate for other sites (Table 2).

**Table 2:** The frequency of pelvic endometriosis according to their localization and imaging modality along with the agreement between magnetic resonance imaging and ultrasonography.

		MRI	US	PABAK (SE)
*Anterior compartment*	Bladder	3	1	0.886 (0.080)
	Endometrioma/hemorrhagic cyst	32	32	Not calculated
*Middle compartment*	Kissing ovary	7	12	0.600 (0.137)
	Hemato-/hydrosalpinx	8	11	0.714 (0.120)
	Round ligament	7	0	Not calculated
*Posterior compartment*	Rectosigmoid colon	25	34	0.486 (0.150)
	Rectovaginal septum	11	0	Not calculated
	Uterosacral ligament	8	2	0.543 (0.144)
*Others*	Adhesion	27	28	0.600 (0.137)
	Ileum	1	0	Not calculated

PABAK: prevalence-adjusted bias-adjusted kappa; SE: standard error; US: ultrasonography; MRI: magnetic resonance imaging.

Specifically, for the rectosigmoid involvement, the length of the lesion was longer on US than on MRI. Detection of DPE on MRI and optimal assessment of its length may not be possible due to intestinal gas. Thickening and obliteration of the rectovaginal septum were observed only on MRI but not on US, which might be due to an inadequately filled or overfilled bladder that makes it difficult to evaluate this structure correctly.

**Figure 1:**
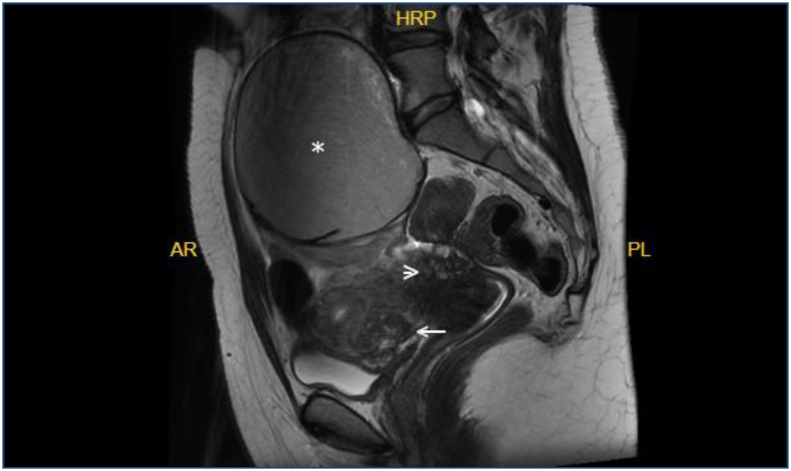
Sagittal T2-weighted imaging. Endometrioma with "T2 shading sign"(asterisk), vesicouterine space and posterior bladder wall endometriosis (arrow), and torus uterinus involvement (arrowhead).

In one case, a focus of endometriosis on the bladder wall extending into the lumen was identified on both US and MRI (Figure 1). The findings are summarized in Table 2.

## DISCUSSION AND CONCLUSION

DPE is characterized by endometriosis tissues, invading surrounding organs and causing inflammatory processes, fibrosis, and adhesions, and distorting the pelvic anatomy^8^. The management of DPE requires a multidisciplinary approach and individualized treatment options^9^. The diagnosis of pelvic endometriosis is based on anamnesis, physical examination, and various imaging methods. Although laparoscopy and histopathologic examination are the gold standard for diagnosing of deep endometriosis, preoperative imaging methods for detection, mapping, and treatment planning are very important. The two most commonly used imaging modalities are US and MRI. The diagnostic performances of US and MRI performed by experienced radiologists for DPE are high^10-12^. MRI is the preferred method for determining the extent of the disease and the size and localization of the implants and for accurate preoperative mapping due to its high soft tissue resolution and image acquisition in different planes^13-15^. The present study compares the agreement between US and MRI, and demonstrates a perfect agreement for endometrioma/hemorrhagic cyst, almost perfect agreement for bladder involvement, and moderate agreement for other sites.

Ovaries are the most commonly involved structure in pelvic endometriosis. In our series, a single or bilateral endometrioma or hemorrhagic cyst was detected in 32 of the 35 patients. A kissing ovary was defined in 12 patients on US and seven patients on MRI. Eleven patients had hydro-/hematosalpinx on US and eight of them were positive on MRI. Being real-time imaging and allowing positional change during imaging to obtain better images may provide a superiority of US over MRI imaging in the assessment of hydro-/hematosalpinx and ovaries.

USL has been reported as the second most common site of involvement after the ovaries^16^. Retroflexed uterus, uterine fibroids, or ovarian cysts may obscure USL. If the USL is not thick or there is no pelvic fluid, it cannot be seen with US^17^. In two meta-analyses, the sensitivity and specificity values of TVUS for the diagnosis of USL endometriosis were 53–64% and 93–97%, respectively^18,19^. In our study, USL involvement reported on MRI was relatively low (23% on MRI). Evaluating the ligaments can be challenging due to tissue distortion and adhesions, which complicate the assessment of normal anatomy. The high degree of adhesions in our cases might have affected the interpretation of these structures. Additionally, the definition of USL involvement, which varies among the studies, may affect the prevalence of this finding^7^.

In clinical practice, involvement of the rectosigmoid colon is common, as in our and torus uterinus involvement often accompanies this finding. In some cases, rectosigmoid wall thickening detected by US was not detected by MRI. The reason for this is the difficulty in differentiating DPE nodules due to the hypointense signal of colonic gas. For the same reason, DPE dimensions can be evaluated more reliably with US. In our study, the length of rectosigmoid DPE was less on MRI than US in all cases. Torus uterinus involvement is rarely reported in TVUS reports due to the difficulty in evaluating the posterior wall of the uterus^20^. It is more difficult to evaluate the posterior uterine wall with US, especially in the presence of a retroverted or retroflex uterus. In our study, torus uterinus involvement was reported in 10 patients on MRI and only in one patient on US.

In two meta-analyses, it was reported that US and MRI showed similar diagnostic performance in demonstrating DPE involvement in the rectosigmoid, rectovaginal septum, and USL^21,22^. In our study, although the agreement of US and MRI was acceptable in demonstrating rectosigmoid DPE, the reporting rate of USL and rectovaginal septum obliteration was significantly lower than that of MRI.

Anterior compartment involvement is rare, and various studies have reported involvement rates between 2 and 8.4%^23^. The vagina and urinary tract were the least frequently reported sites of involvement (0.9 and 0.7%, respectively). In a meta-analysis, the sensitivity and specificity of TVUS in detecting vaginal fornix involvement were 57 and 99%, respectively^24^. In our study, rectovaginal involvement was observed in 11 patients on MRI. The use of vaginal gel may provide better visualization of vaginal involvement on MRI.

Bladder involvement is very rare. Sensitivity and specificity for bladder endometriosis have been reported as 55 and 93.5%, respectively^25^. Involvement of the bladder and prevesical space was identified on MRI in three patients, and bladder involvement was pathologically confirmed in one case in our study. Prevesical space involvement was not reported on US. Small implants in this area may be missed on TVUS.

The limitation of our study was the lack of surgical and pathology results for our cases. In addition, the patients included in the study were those with positive endometriosis findings on US. The strengths of the study are that US and MRI evaluations were performed by experienced radiologists and performed with a strict application of the guidelines.

In conclusion, identifying and defining the affected localizations of DPE and its sequelae before diagnostic and therapeutic surgery are crucial. In the present study, we found perfect agreement for endometrioma/hemorrhagic cyst, almost perfect agreement for bladder involvement, and moderate agreement for other sites, and the possible reasons for the disagreements were discussed. US and MRI have their own advantages and disadvantages in diagnosing DPE. Due to the lack of real-time visualization on MR, hydro-/hematosalpinx may not be differentiated from a septated complicated cyst. US has limitations such as a limited field of view, difficulty in visualizing some structures, and operator dependency. Evaluation of the posterior uterine wall on US may be difficult. As the agreement varies according to the sites of involvement, US and MRI are not standalone diagnostic techniques, and should be considered complementary imaging methods. In addition to assessing patients with suspected DPE in line with the guidelines, paying attention to the potential diagnostic issues highlighted in our study will increase the likelihood of accurate diagnosis.
